# *Campylobacter jejuni* Virulence Factors Identified by Modulating Their Synthesis on Ribosomes With Altered rRNA Methylation

**DOI:** 10.3389/fcimb.2021.803730

**Published:** 2022-01-13

**Authors:** Agnieszka Sałamaszyńska-Guz, Pernille Kronholm Rasmussen, Małgorzata Murawska, Stephen Douthwaite

**Affiliations:** ^1^ Division of Microbiology, Department of Pre-Clinical Sciences, Institute of Veterinary Medicine, Warsaw University of Life Sciences – SGGW, Warsaw, Poland; ^2^ Department of Biochemistry and Molecular Biology, University of Southern Denmark, Odense, Denmark

**Keywords:** *Campylobacter jejuni*, rRNA methylation, TlyA, OMV, MlaEFD, CDT

## Abstract

*Campylobacter jejuni* is a major cause of food poisoning worldwide, and remains the main infective agent in gastroenteritis and related intestinal disorders in Europe and the USA. As with all bacterial infections, the stages of adhesion to host tissue, survival in the host and eliciting disease all require the synthesis of proteinaceous virulence factors on the ribosomes of the pathogen. Here, we describe how *C. jejuni* virulence is attenuated by altering the methylation of its ribosomes to disrupt the composition of its proteome, and how this in turn provides a means of identifying factors that are essential for infection and pathogenesis. Specifically, inactivation of the *C. jejuni* Cj0588/TlyA methyltransferase prevents methylation of nucleotide C1920 in the 23S rRNA of its ribosomes and reduces the pathogen’s ability to form biofilms, to attach, invade and survive in host cells, and to provoke the innate immune response. Mass spectrometric analyses of *C. jejuni* TlyA-minus strains revealed an array of subtle changes in the proteome composition. These included reduced amounts of the cytolethal distending toxin (CdtC) and the MlaEFD proteins connected with outer membrane vesicle (OMV) production. Inactivation of the *cdtC* and *mlaEFD* genes confirmed the importance of their encoded proteins in establishing infection. Collectively, the data identify a subset of genes required for the onset of human campylobacteriosis, and serve as a proof of principle for use of this approach in detecting proteins involved in bacterial pathogenesis.

## Introduction

*Campylobacter jejuni* infections are the leading cause of human bacterial gastroenteritis in developed countries, and are characterized by attachment and invasion of the colonic epithelium leading to inflammation of the bowel and diarrheal disease. In severe cases, symptoms can progress to septicemia, post-infectious arthritis, Guillain-Barré syndrome (Yuki et al., 2004), Miller Fisher syndrome (Ang et al., 2002), or chronic bowel disorders such as Crohn’s disease and ulcerative colitis (Johnson et al., 2017). Provoking this range of pathogenic effects undoubtedly requires the coordinated expression of a series of *C*. *jejuni* genes that are specifically dedicated to virulence. For instance, this pathogen must first survive the handling and cleaning processes such as those involved in the slaughter of chickens and the preparation of meat products (Hermans et al., 2011; Rasschaert et al., 2020); then, after consumption, the pathogen must remain viable in the human gut to initiate the steps of colonization; and subsequently, when established, the visible symptoms of gastrointestinal disease ensue upon production of virulence factors including toxins (Kreling et al., 2020; Elmi et al., 2021). The molecular mechanisms that direct these steps in *C*. *jejuni* pathogenesis are presently largely unknown. We address this problem here using an approach that modulates the expression of subsets of *C. jejuni* genes, enabling the identification of factors that are necessary for its virulence.

The infectious properties of bacteria pathogens are dependent on the expression of proteinaceous virulence factors on their ribosomes. The process of protein expression is fine-tuned by sets of modified nucleotide residues within the RNA components of the ribosome (Purta et al., 2009; Sergiev et al., 2018). We have previously reported that loss of a single methyl group modification on the ribose of nucleotide C1920 of *C. jejuni* 23S rRNA severely attenuates the pathogen’s virulence (Sałamaszyńska-Guz et al., 2018). Modification of nucleotide C1920 is catalyzed by the methyltransferase Cj0588, and inactivation of this enzyme results in a wide range of defects including reduced ribosome subunit association, biofilm formation, invasion of epithelial cells, and diminished survival of *C. jejuni* in macrophages (Sałamaszyńska-Guz et al., 2018; Sałamaszyńska-Guz et al., 2020). The *C. jejuni* Cj0588 enzyme is homologous to the methyltransferase TlyA^I^ found in certain other bacterial species, where it modifies the same rRNA nucleotide and has also been linked with a range of virulence traits (Monshupanee et al., 2012; Monshupanee, 2013; Keith et al., 2021).

Here, a set of *C. jejuni* strains defective in nucleotide C1920 methylation are used to identify key genes that are specifically linked with virulence. We have identified most of the potential proteins within the *C. jejuni* proteome using mass spectrometry, and show that significant downregulation of a limited subset of genes coincides with loss of virulence. Downregulated genes include those encoded the cytolethal distending toxin complex (CDT), and genes of the *mlaEFD* operon that are necessary for maintaining the asymmetry of the outer membrane (OM) and consequently for outer membrane vesicle (OMV) production. Knockouts of the *cdtC* and *mlaEFD* genes were constructed and their effects on biofilm formation (required for *C. jejuni* survival and transmission in food preparation), epithelial cell attachment and invasion (required for gut colonization), and cytokine stimulation and survival in macrophages (required for disease progression) were followed and compared to a collection of TlyA^+^ and TlyA^-^ strains. Collectively, the data home in on a limited collection of genes linked with human campylobacteriosis.

## Materials and Methods

### Bacterial Strains

The *C. jejuni* strains used in this study (**Table 1**) were grown under microaerobic conditions (BD GasPak EZ CO_2_ sachets, Becton Dickinson) at 37°C on brain-heart infusion (BHI) agar containing 5% (v/v) sheep blood, supplemented when necessary with chloramphenicol at 20 µg ml^-1^.

**Table 1:** *C. jejuni* strains used in this study.

Strains	Relevant characteristics	Source/reference
*C. jejuni* 81-176	Wild-type (WT)	Korlath et al., 1985
*C. jejuni* 81-176 Δ*tlyA*	Cm^r^, *tlyA (cj0588)* deletion mutant	Sałamaszyńska-Guz et al., 2018
*C. jejuni* 81-176 Δ*tlyA*::*tlyA*	Cm^r^, Km^r^, *tlyA* deleted, complemented with wild-type *C. jejuni tlyA*	Sałamaszyńska-Guz et al., 2018
*C. jejuni* 81-176 Δ*tlyA*::*tlyA*K188A	Cm^r^, Km^r^ *tlyA* deleted, complemented with *tlyA* mutated at K188A	Sałamaszyńska-Guz et al., 2018
*C. jejuni* 81-176 Δ*cdtC*	Cm^r^, *cdtC (cj0077)* deletion mutant	This study
*C. jejuni* 81-176 Δ*mlaEFD*	Cm^r^, *mlaE (cj1646), mlaF (cj1647)* and *mlaD (cj1648)* deletion mutant	This study

The generic notation (tlyA) is used throughout the text for the C. jejuni gene cj0588 encoding the methyltransferase that modifies the 23S rRNA nucleotide C1920.

### Protein Extraction and Peptide Labeling

Overnight cultures of *C. jejuni* strains were harvested by centrifugation (Sałamaszyńska-Guz et al., 2018) and resuspended in lysis buffer (7 M urea, 2 M thiourea, 1% DTT, and 0.5% n-octyl-β-D-glucopyranoside) supplemented with protease inhibitors (Roche), and were ultrasonicated for protein extraction. After measuring the protein concentrations (Bradford reagent, Merck), 200 µg samples were alkylated with 45 mM iodoacetamide (IAA) incubated for 30 min in the dark before adding 5 mM DTT and adjusting to pH 8.5 with 30 mM triethylammonium bicarbonate (TEAB) buffer. Proteins were predigested for 3 h at 37°C with 0.005 AU lysyl endopeptidase (Lys-C, FUJIFILM Wako Pure Chemical Corporation). Samples were then further digested at 37°C with methylated trypsin at an enzyme:protein ratio of 1:50 (Heissel et al., 2018) prior to quenching with 10% trifluoroacetic acid (TFA) and centrifuging at 20,000 g for 15 min. Peptides were desalted on R2C8 and R3C18 resin reversed-phase columns (Applied Biosystems, POROS 20 R2; Thermo Fisher Scientific), washed with 0.1% TFA and eluted with 60% acetonitrile/0.1% TFA before drying under vacuum centrifugation and resuspending in 50 mM TEAB buffer.

The peptides were labelled with isobaric tags (iTRAQ 8-plex™, AB SCIEX) according to the supplier’s recommendations; equal amounts of the samples were pooled and acidified prior to desalting on reverse-phase columns. Peptides derived from wild-type cells were tagged with iTRAQ-114 and iTRAQ-117; Δ*tlyA* peptides with iTRAQ-115 and iTRAQ-118; Δ*tlyA*::*tlyA* with iTRAQ-116 and iTRAQ-119; and Δ*tlyA*::*tlyA*K188A with iTRAQ-117 and iTRAQ-121.

### LC-MS/MS Tandem Mass Spectrometry

The iTRAQ-labeled peptides were fractionated by reversed-phase chromatography on a Dionex UltiMate 3000 HPLC^+^ Focused system (Thermo Fisher Scientific) equipped with an ACQUITY UPLC M-class, CSH C18 130 Å, 1.7 µm, 300 µm ×100 mm analytical column (Waters). Mobile phase A comprised 20 mM ammonium formate in water, pH 9.3; phase B was 80% acetonitrile and 20% solvent A, pH 9.3. Peptides were eluted at a flow rate of 5 µL/min over 71 min in a gradient of solvent B rising from 2% to 40%, followed by 50% solvent B to 122 min, before finally washing with 95% solvent B for 10 min. Twenty fractions were collected for each peptide set.

Fractions were separated on a C18 trap column (100 µm ID × 3 cm, 5 µm, Reprosil-Pur 120 C18-AQ, Dr. Maisch GmbH) and a C18 analytical column (75 µm ID × 18 cm, 3 µm, Reprosil-Pur 120 C18-AQ, Dr. Maisch GmbH) at a flow rate of 250 nL/min in a multi-step gradient of 5% BMS buffer (95% acetonitrile, 0.1% formic acid) raised to 10% BMS over 5 min, to 34% BMS from 5 to 125 min, to 50% BMS from 125 to 135 min, and finally washed with 100% BMS. The EASY-nLC was connected online to a Q Exactive HF mass spectrometer (Thermo Fisher Scientific). MS analyses were performed in data-dependent acquisition mode at a resolution of 120,000 (*m/z* 200) with the mass range of 350-1600 *m/z*. The target values were set to 3.00E+06 with a maximum injection time of 100 ms. The fifteen most abundant ions were selected from the MS with an isolation width of 1.2 *m/z* for fragmentation in the HCD collision cell using a collision energy of 34%. Fragmentation was at a resolution of 30,000 (*m/z* 200) for a target value of 1.00E+05 with a maximum injection time of 120 ms (Thingholm et al., 2010).

### Protein Identification and Quantification

Raw data files were processed by Thermo Proteome Discoverer software v 2.4 (Thermo Fisher Scientific). Protein identification was made from the *C. jejuni* 81-176 genome sequence (Uniprot databases) using the Sequest search engine. For the search parameters: precursor mass tolerance was set to 10 ppm, with a fragment mass tolerance of 0.02 Da; carbamidomethylation of cysteine, iTRAQ modification of N-termini and of lysine residues were added to dynamic modifications, while acetylation was set to variable modifications; two missed cleavage sites per protein were allowed. For reporter ion quantification, the co-isolation threshold was set to 50, and average reporter ion S/N threshold to 10. Data from Proteome Discoverer was processed using Excel (Microsoft).

Quantification was based on three biological replicates of each strain, and taking into consideration only the proteins that were identified from at least two unique peptides and with a false discovery rate of less than 1% (Sun et al., 2020; Orsburn, 2021). The abundances of individual proteins that changed ≥ 1.5-fold with *p*-values below 0.05 (Cottrell, 2011) were considered as being expressed to a significantly different degree in the comparisons of the wild-type with the Δ*tlyA* strain, the wild-type with the *ΔtlyA::tlyA*K188A strain, the *ΔtlyA* with the Δ*tlyA*::*tlyA* strain, and the Δ*tlyA*::*tlyA* with the *ΔtlyA::tlyA*K188A strain.

### Construction of *C. jejuni* Mutants Strains

A single-gene knockout of the *C. jejuni* 81-176 strain was created by inserting a *cat* chloramphenicol resistance cassette (Cm^r^) into the *cdtC* gene. Briefly, the gene was amplified by PCR using the cdtCR and cdtCF primers (all primers are listed in **Table S1**) and cloned into the pJET1.2 vector (ThermoFisher Scientific), prior to insertion of the *cat* cassette using the primers cdtC_mutR and cdtC_mutF, replacing 12 bp of the *cdtC* gene and disrupting its coding sequence. The plasmids lack a *C. jejuni* replicon and, after electrotransformation into *C. jejuni* cells, retention of Cm^r^ thus requires allelic exchange with the homologous chromosomal sequence. The null-mutant *C. jejuni* 81-176 Δ*cdtC* (**Table 1**) was used in subsequent experiments. The *C. jejuni* 81-176 Δ*mlaEFD* mutant lacking all three of these genes was constructed in a similar manner using the primers 1637F with 1639R, and then 1637_mutR with 1639_mutF. In each of the knockout strains, *cat* was inserted into the chromosome the same transcriptional direction as the inactivated gene (*tlyA*, *cdtC* or *mlaEFD*). All constructs were verified by PCR sequencing at each stage of the process.

### Virulence Phenotypes

Assays to assess biofilm formation ability of the *C. jejuni cdt* and *mla* strains, as well as their adhesion and invasion of Caco-2 epithelial cells, and the immune responses elicited in T84 epithelial cells were performed as previously described for the *tlyA* strains (Sałamaszyńska-Guz et al., 2020).

### Isolation of Outer Membrane Vesicles (OMVs)

OMV were prepared from by filtering overnight cultures through a 0.2 µm-pore filter and passing the supernatants through 10 kDa centrifugal concentrators (Thermo Fisher Scientific) followed by the ExoBacteria™ OMV Isolation Kit (System Biosciences). The purity of OMV samples was ascertained by transmission electron microscopy (TEM), and their protein content was measured spectrophotometrically based on the Bradford method (Zingl et al., 2020). In the adhesion and invasion assays, Caco-2 cells and the wild-type *C. jejuni* strain (MOI = 100) were co-incubated with OMVs (10 μg). The level of IL-8 secretion in T84 cells after co-incubation with 100 μg OMVs was assessed using ELISA.

## Results

### Loss of Methylation at 23S rRNA Nucleotide C1920 Changes the *C. jejuni* Proteome

Previously, we reported that loss of TlyA methylation at nucleotide C1920 in 23S rRNA attenuates several virulence traits of *C. jejuni* (**Table S2**); in the present study, the proteomes of *tlyA*-defective *C. jejuni* strains have been analyzed to identify proteins that are linked with virulence. The genome of *C. jejuni* 81-176 together with the strain’s two resident plasmids encode 1813 open reading frames (ORFs), and using a mass spectrometry-based quantitative proteomic approach we detected 1482 of the protein products (1424 encoded by the chromosome, 35 by plasmid pVir, and 23 by plasmid pTet), representing 82% of the potential proteome. Applying the criteria described in the methods section, the levels of 50 proteins were found to differ significantly in the *tlyA* null-mutant (lacking C1920 methyltransferase) compared to the wild-type strain, with 27 proteins upregulated and 23 downregulated (**Tables 2** and **S3**). Amongst the upregulated proteins, eleven chromosomally encoded sequences are involved in the assembly and modification of flagella, six are engaged in amino acid biosynthesis, one (CstA) in carbon starvation, one (DnaJ) as a molecular chaperone, and five are not yet characterized. The three remaining proteins are encoded by pVir. The downregulated proteins included five enzymes associated with lipid turnover, four ribosomal proteins, three proteins involved in oxidative phosphorylation, two members of the acetyltransferase GNAT family, proteins associated with ATP production, protein secretion, transport of lipopolysaccharides and capsular polysaccharides, transferase of ions, iron uptake, one toxin-like protein, and one protein that remains uncharacterized (**Tables 2** and **S3**).

**Table 2:** Significant changes in the proteome of the *C. jejuni ΔtlyA* strain compared with the wild-type strain.

Proteins with enhanced expression in the Δ*tlyA* strain	Gene ID	Proteins with reduced expression in the Δ*tlyA* strain	Gene ID
Uncharacterized protein	CJJ81176_0206	Cpp4	CJJ81176_pTet0052
Uncharacterized protein	CJJ81176_0207	Cytochrome c oxidase, cbb3-type, subunit I, CcoN	CJJ81176_1482
Uncharacterized protein	CJJ81176_0078	Thioesterase family protein	CJJ81176_0922
Flagellin	CJJ81176_1339	Cytochrome c553, Cyf	CJJ81176_1170
Uncharacterized protein	CJJ81176_1458	50S ribosomal protein L35	CJJ81176_0269
Lipoprotein, putative	CJJ81176_1045	Acetyltransferase, GNAT family	CJJ81176_0985
Flagellin modification protein, PseA	CJJ81176_1333	Uncharacterized protein	CJJ81176_1104
Anthranilate synthase component I, TrpE	CJJ81176_0369	50S ribosomal protein L34	CJJ81176_0984
Basal-body rod modification protein, FlgD	CJJ81176_0080	ATP synthase subunit c, AtpE	CJJ81176_0943
Flagellar basal-body rod protein, FlgG	CJJ81176_0721	Lipoprotein, putative	CJJ81176_1640
Flagellin	CJJ81176_1338	Acetyltransferase, GNAT	CJJ81176_0250
N-(5’-phosphoribosyl)anthranilate isomerase, TrpF	CJJ81176_0371	Sodium/hydrogen exchanger family protein	CJJ81176_1245
Uncharacterized protein	CJJ81176_1344	Preprotein translocase, YajC	CJJ81176_1112
Para protein, Cjp26	CJJ81176_pVir0025	50S ribosomal protein L28	CJJ81176_0475
Tryptophan synthase alpha chain, TrpA	CJJ81176_0373	Putative sugar transferase	CJJ81176_1435
Carbon starvation protein A, CstA	CJJ81176_0924	Cytolethal distending toxin, subunit C, CdtC	CJJ81176_0114
Flagellar P-ring protein, FlgI	CJJ81176_1455	Protein TonB	CJJ81176_1621
Uncharacterized protein, Cjp48	CJJ81176_pVir0048	ABC transporter, ATP-binding protein, MlaF	CJJ81176_1638
Flagellar hook-associated protein, FlgK	CJJ81176_1459	50S ribosomal protein L33	CJJ81176_0500
Flagellar L-ring protein, FlgH	CJJ81176_0710	ABC transporter, periplasmic substrate-binding protein, MlaD	CJJ81176_1639
Uncharacterized protein, Cjp27	CJJ81176_pVir0026	OstA family protein, LptA	CJJ81176_0677
Anthranilate phosphoribosyltransferase, TrpD	CJJ81176_0370	ABC transporter, permease protein, MlaE	CJJ81176_1637
Uncharacterized protein	CJJ81176_1429	Hydrogenase, (NiFe)/(NiFeSe) small subunit family	CJJ81176_1398
UDP-N-acetylglucosamine 4,6-dehydratase, FlmA	CJJ81176_1310		
Tryptophan synthase beta-chain, TrpB	CJJ81176_0372		
Putative imidazole glycerol phosphate synthase subunit, HisF-1	CJJ81176_1331		
DnaJ domain protein	CJJ81176_1053		

This list of proteins was narrowed considerably to a smaller group directly related to virulence by comparison of the methyltransferase-deficient strain ΔtlyA::tlyAK188A with the isogenic, methyltransferase-active strain ΔtlyA::tlyA (**Table 3**). The degrees of change in protein expression for all strain comparisons are listed in **Table S3**.

Presumably, several of these changes in protein abundance are not be directly related to bacterial virulence, but instead result from other causes such as the introduction of the chloramphenicol resistance cassette into the *tlyA* gene. The proteome analyses were therefore repeated to include two isogenic strains, the first of which was the *tlyA* null-mutant complemented with an active copy of *tlyA* (which restores virulence), and the second was the null-mutant complemented with a methyltransferase-deficient copy of *tlyA* [the K188A variant, which does not restore virulence (Sałamaszyńska-Guz et al., 2018)]. Comparison of the proteomes of these two strains, which are genetically identical except for the change at the *tlyA*-188 allele, showed the number of significant changes was limited to eight proteins (**Table S3**). For the purpose of further analysis, this list was narrowed down to the four proteins that were consistently downregulated in all the different combinations of TlyA^+^ versus TlyA^-^ strain comparisons (**Table 3**).

**Table 3:** Significant differences in the proteome of *C. jejuni* in the various TlyA^+^ versus TlyA^-^ strain comparisons.

UniProt ID	Description	Gene Symbols		Abundance ratios			
		81-176 strain	NCTC11168 strain	Δ*tlyA* vs. WT	Δ*tlyA* vs. Δ*tlyA::*Δ*tlyA*	Δ*tlyA::*Δ*tlyA*K188A vs. WT	Δ*tlyA*::*tlyA*K188A vs. Δ*tlyA*::*tlyA*
A0A0H3PCP5	Cytolethal distending toxin, subunit C, CtdC	CJJ81176_0114	*cj0077*	0.449	0.548	0.394	0.462
A0A0H3PA07	ABC transporter, putative permease protein, MlaE	CJJ81176_1637	*cj1646*	0.320	0.409	0.335	0.433
A0A0H3PE90	ABC transporter, ATP-binding protein, MlaF	CJJ81176_1638	*cj1647*	0.429	0.588	0.376	0.500
A0A0H3PBI5	ABC transporter, putative periplasmic substrate-binding protein, MlaD	CJJ81176_1639	*cj1648*	0.394	0.580	0.349	0.576

Proteins with a consistent fold change ≥ 1.5 in three biological replicates were considered as differentially-expressed proteins in the ΔtlyA strain compared to the wild-type (WT), the ΔtlyA strain versus ΔtlyA::tlyA, the ΔtlyA::tlyAK188A strain versus WT, and the strain ΔtlyA::tlyAK188A versus ΔtlyA::tlyA. The UniProt ID is given for each of the proteins together with the generic protein designation (e.g. CdtC) and the corresponding strain-specific gene notations (e.g. CJJ81176_0114 for the C. jejuni 81-176 strain; and cj0077 for the NCTC11168 strain). A comprehensive list of all the significant changes is given in **Table S2**, and includes other proteins potentially involved in pathogenesis. However, the four downregulated proteins listed here were the only proteins to show consistent abundance changes in all of the different TlyA^+^ versus TlyA^-^ strain comparisons.

**Figure 1:**
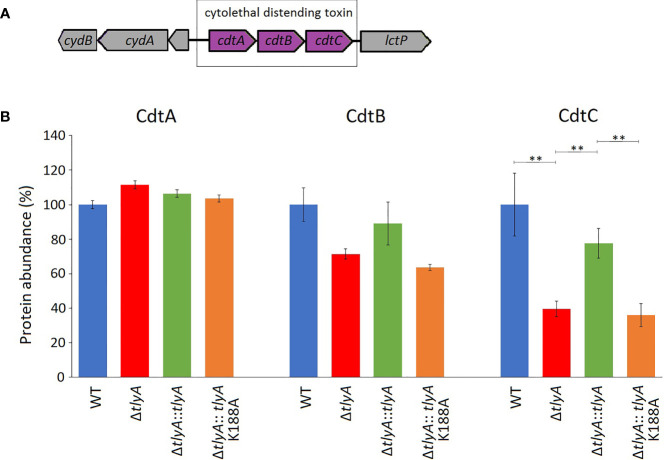
Changes in the abundance of CdtABC proteins upon inactivation of the *tlyA* gene. **(A)** Organization of the *cdtABC* gene cluster in *C. jejuni*. The strain-specific notations for these genes are given in . **(B)** Abundances of the individual Cdt proteins in the wild-type (WT) *C. jejuni* strain, the *tlyA* knockout strain (Δ*tlyA*), and the knockout strain complemented with active (Δ*tlyA::tlyA*) and inactive (Δ*tlyA::tlyA K188A*) copies of the *tlyA* gene. The protein amounts in the wild-type strain have been normalized here to 100%. The abundance of CdtC is dependent on a functional *tlyA* gene; CdtB is also reduced (but outside the significance cut-off applied here); and the upstream encoded protein CdtA remained unchanged. The error bars represent the standard deviations of three biological replicates; **P < 0.001 for these three strain comparisons.

One of these proteins, CdtC, interacts with the subunits CdtA and CdtB to form the cytolethal distending toxin (CDT). In *C. jejuni* strains lacking the TlyA modification of the rRNA, the abundance of CdtC protein was reduced to 39% and CdtB to 71% of the wild-type levels (outside the cut-off applied here), while no change was seen in the level of CdtA (**Figure 1**). Complementation with the active copy of *tlyA* restored abundance of CdtC and CdtB proteins to 77% and 89% of wild-type levels, respectively. No rescue occurred with the methyltransferase-defective K188A version of TlyA.

**Figure 2:**
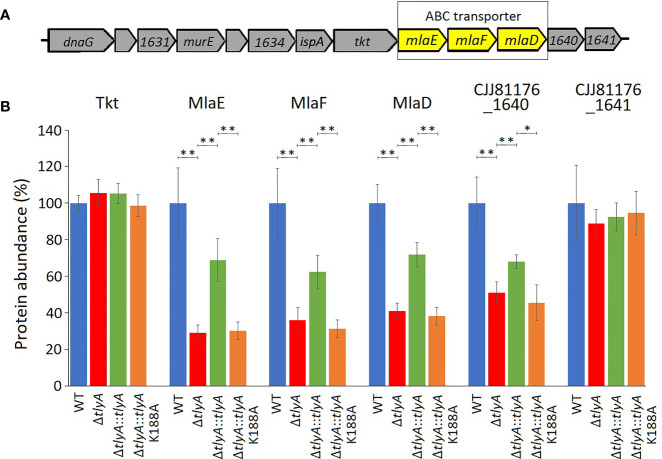
Changes in abundances in the proteins encoded within and adjacent to the *C. jejuni mlaEFD* operon upon inactivation of *tlyA* (color-coded as in **Figure 1**). **(A)** Organization of the *C. jejuni mlaEFD* gene cluster. The strain-specific notations for these genes are given in **Table 3**. **(B)** The abundance of each of the MlaE, MlaF and MlaD proteins is dependent on a functional *C. jejuni tlyA* gene **P < 0.001 (strain notation as in **Figure 1**). The abundances of the upstream-encoded protein Tkt remained unchanged. However, expression of the downstream-encoded protein CJJ81176_1640 (**Table 2**, and hereafter referred to as CJ1640) was consistently reduced, *P < 0.05, (but outside our cut-off criteria), while its CJ1641 neighbor was unchanged. These three additional proteins are shown here for comparison. The error bars represent standard deviations from three biological replicates.

The other three downregulated proteins are encoded by the *mla* gene cluster (**Figure 2**), and are homologs of MlaE, MlaF and MlaD that comprise an *E. coli* ABC-transporter with which they show 28, 32, and 19% respective similarity in their amino acid sequences. In the *C. jejuni* Δ*tlyA* strain, the abundances of MlaE, MlaF and MlaD were respectively reduced to 29, 36, and 41% of the wild-type levels. Expression of *CJJ81176_1640* (**Table 2**, and hereafter referred to as *cj1640*), the next gene downstream, was also lowered by *tlyA* inactivation, albeit to a lesser degree than *mlaEFD*; however, expression of *tkt* and *cj1641* immediately upstream and downstream of these genes remained unaffected (**Figure 2**). The abundances of MlaE, MlaF, MlaD and CJ1640 were partially restored to around two-thirds of the wild-type levels upon complementation with an active copy of *tlyA* (**Figure 2**); again, no rescue occurred with the methyltransferase-defective K188A version of this gene.

### Inactivation of *cdtC* and *mlaEFD* Decreases the Virulence of *C. jejuni*

**Figure 3:**
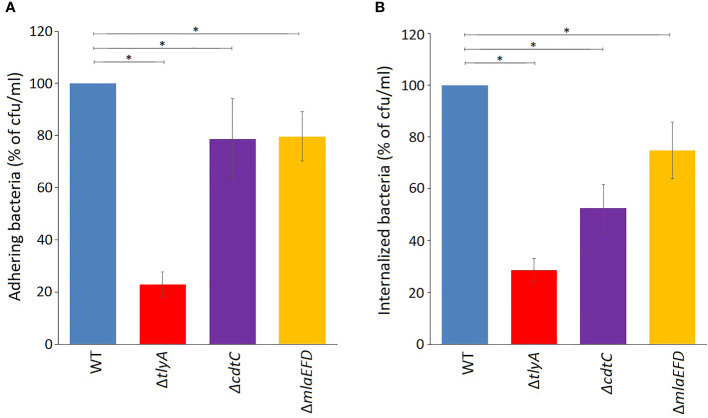
Attenuation of virulence properties in the *C. jejuni* mutant strains. **(A)** Adhesion onto Caco-2 epithelial cells by *C. jejuni* was significantly reduced by the mutations; *P < 0.05 for Δ*tlyA*, Δ*cdtC* and Δ*mlaEFD* versus the wild-type (WT). **(B)** Invasion of *C. jejuni* into Caco-2 epithelial cells was also significantly reduced by each of these mutations; *P < 0.05 for Δ*tlyA*, Δ*cdtC* and Δ*mlaEFD* versus the wild-type (WT). Values represent means ± S.E.M. of three independent experiments.

The lower amounts of the CdtC and MlaEFD proteins correlate with reduced ability of the *C. jejuni* TlyA-minus strains to adhere, invade and induce IL-8 secretion in host cells (**Table S2**). These observations were tested further by inactivating *cdtC* and the *mlaEFD* gene cluster and reassessing pathogenicity traits. Loss of either CdtC or the MlaEFD proteins decreased bacterial adherence to Caco-2 host cells to the same degree (around 78% of the wild-type strain, **Figure 3A**). A different picture emerged regarding the invasive properties of the strains, where loss of *cdtC* elicited a more marked effect that inactivation of the *mlaEFD* gene cluster (**Figure 3B**). It should be noted that neither of these deletion mutants adversely affected adherence and invasion to the same extent as the *tlyA* deletion (**Figures 3A, B**).

Other parameters used to evaluate the health of *C. jejuni* strains, such as growth rates and the ability to form biofilms, indicated that these criteria were not adversely affected by deletion of the *cdtC* gene (not shown). However, we saw indications that loss of the *mlaEFD* operon has a potential health cost. While the growth of static cultures and the formation of biofilms by the *mlaEFD* strain were not adversely affected, agitation of cultures during growth lowered the viable cell count by approximately 30% compared to that of the wild-type. In addition, loss of *mlaEFD* increased the susceptibility of *C. jejuni* to polymyxin antibiotics, whereas no discernible effect was seen upon *tlyA* inactivation (**Table S4**). Neither of the mutations changed vancomycin susceptibility.

### The Influence of *C. jejuni* OMVs on Adherence and Invasion of Epithelial Cells

**Figure 4:**
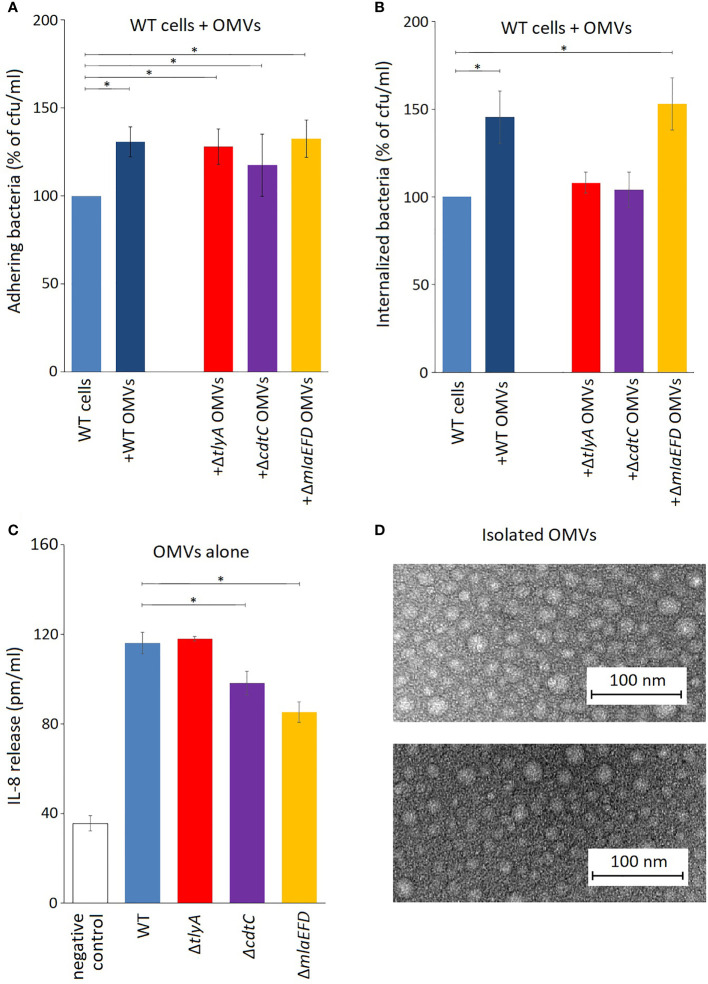
OMVs produced by *C. jejuni* strains. **(A)** Influence of *C. jejuni* OMVs on adhesion to epithelial cells. Purified OMVs (10 μg) from the various strains were added to viable wild-type *C. jejuni* and incubated for 2 h with Caco-2 cells. Addition of OMVs isolated from wild-type and mutant strains significantly increased adhesion of *C. jejuni* to Caco-2 cells (*P < 0.05). **(B)** OMVs from the wild-type and Δ*mlaEFD* strains (added and incubated as in panel A) significantly improved internalization of wild-type *C. jejuni* into Caco-2 epithelial cells (*P < 0.05). No significant change in *C. jejuni* internalization was observed with OMVs from the Δ*tlyA* or Δ*cdtC* strains. **(C)** Secretion of interleukin IL-8 by T84 cells during incubation with 100 μg OMVs from the various strains, in the absence of viable *C. jejuni* cells. Significantly less IL-8 was produced upon addition of OMVs from the Δ*cdtC* and Δ*mlaEFD* strains compared to wild-type OMVs (*P < 0.05); however, no significant difference between wild-type and Δ*tlyA* OMVs was observed. **(D)** Representative images of purified OMVs from the *C. jejuni* wild-type strain (upper panel) and Δ*mlaEFD* (lower panel) visualized by transmission electron microscopy. No visible differences were observed between OMVs that were isolated from wild-type strain and mutants. The OMV populations, purified by the resin-binding procedure used here, had a size distribution of between 10 to 30 nm in diameter.

The outer membrane vesicles (OMVs) of *C. jejuni* play an important role in its ability to adhere to and invade epithelial cells (Elmi et al., 2012). These pathogenic properties of *C. jejuni* are enhanced by co-incubation with an extra portion of isolated OMVs (**Figures 4A, B**). Addition of OMVs from the *tlyA*, *cdtC* and *mlaEFD* mutants also improved the adhesion of the *C. jejuni* wild-type strain to Caco-2 epithelial cells (**Figure 4A**). Bacterial internalization into epithelial cells was also improved by addition of OMVs from the wild-type and *mlaEFD* strains (**Figure 4B**). However, coincubation with extra OMVs derived from the *tlyA* and the *cdtC* mutants did not improve internalization of *C. jejuni*.

Production of interleukin-8 (IL-8) by T84 epithelial cells was assayed using ELISA to measure the induction of an innate immune response. Addition of OMVs from all the *C. jejuni* strains elicited release of IL-8, although the stimulatory effect was significantly lower with OMVs from the *cdtC* the *mlaEFD* mutant strains (**Figure 4C**).

## Discussion

Loss of some rRNA modifications have been reported to cause a series of pleiotropic effects, presumably due to changes in protein expression, and can result in reduced virulence in pathogenic bacteria (Sergiev et al., 2018). One example of such effects is brought about by inactivation of the methyltransferase TlyA^I^, which 2´-*O*-methylates nucleotide C1920 in 23S rRNA (Monshupanee et al., 2012). Loss of this modification in *C. jejuni* rRNA through inactivation of its TlyA^I^ homolog Cj0588 results in wide-ranging defects in ribosome subunit association, cell motility, biofilm formation, adherence to and invasion of human epithelial cells, and survival within macrophages (Sałamaszyńska-Guz et al., 2018; Sałamaszyńska-Guz et al., 2020). The defective ribosomal subunit association is readily explained by loss of rRNA methylation at the site of subunit interaction (Johansen et al., 2006), although the mechanisms behind the extensive reduction in virulence traits (**Table S2**) are more complex. We previously proposed that the loss of the ribosome modification interfered with in protein expression (in manner that remained to be defined), and this in turn resulted in attenuation of bacterial virulence (Sałamaszyńska-Guz et al., 2018).

The present study addresses this idea using a mass spectrometric approach to determine what changes occur in the *C. jejuni* proteome after loss of the Cj0588-catalyzed ribosome modification. Using the stringency criteria described above, the abundances of fifty proteins were significantly different in the *tlyA* mutant compared with the wild-type strain (**Tables 2** and **S3**). The roles of many of these proteins (including, for example, those involved in the assembly of flagella, the formation lipids, lipopolysaccharides and membranes, and the secretion of toxins) could readily be imagined as being linked to pathogenesis. On the other hand, some proteins (such as enzymes for amino acid biosynthesis and oxidative phosphorylation) are probably only indirectly linked with pathogenesis. Some of the remaining proteins undoubtedly have no connection with the loss of *C. jejuni* virulence, but rather result from the process used to disrupt the *tlyA* gene or (in the case of the ribosomal proteins L28, L33, L34 and L35) as a consequence of effects on 50S assembly (Röhl and Nierhaus, 1982) or its association with the 30S ribosomal subunit (Sałamaszyńska-Guz et al., 2018).

The proteome analyses were repeated to include two isogenic strains, the first of which was the *tlyA* null-mutant complemented with an active copy of *tlyA* (which restores the virulence phenotype), and the second was the null-mutant complemented with a methyltransferase-deficient copy of *tlyA* (the K188A variant, which does not restore virulence). Comparison of the proteomes of these two strains, which are genetically identical except for the change at the *tlyA*-188 allele, limited the changes to only a few proteins. Each of these proteins was downregulated in the K188A variant, and differed to approximately the same degree as in the comparison of Δ*tlyA* versus wild-type (**Table S3**). An array of proteins involved in flagellum construction (including FlgD, FlgG, FlgH, FlgI and FlgK) and the structural flagellins were significantly upregulated after inactivation of *tlyA* (**Table S3**). All these proteins were, however, expressed at this same higher level in the *tlyA* strains after complementation with active and inactive copies of this gene, indicating that flagellar protein overexpression was not linked to rRNA methylation but rather to other factors, such as the intrusion of the resistance cassette at the *tlyA* locus. Other candidates that were downregulated to a slightly lesser degree in the K188A variant, including ribosomal proteins such as L28, and were dropped in the final analyses for reasons discussed above.

A limited number of candidates were chosen for further genetic analyses to test whether our approach of altering rRNA methylation is in fact a reliable means of identifying bacterial virulence factors. Here, we included only proteins that were consistently significantly different in each of the TlyA^+^ versus TlyA^-^ strain comparisons (**Table S3**). Thus, four proteins made the final cut: CdtC, MlaE, MlaF and MlaD, all of which were downregulated (**Table 3**). The CdtC protein together with CdtA and CdtB constitute the cytolethal distending toxin (CDT), the components of which are encoded by three contiguous genes *cdtA*, *cdtB*, *cdtC* (Pickett et al., 1996) within a single operon (**Figure 1A**). The CdtB component, whose abundance is also reduced in the *tlyA* mutants (albeit to a lesser degree), appears to be the cytotoxic component of CDT acting through type I deoxyribonuclease (DNase I) activity to cause DNA breakage at the G2/M phase in epithelial host cells, while the CdtA and CdtC proteins serve as carriers for delivering CdtB subunit across the host cell membrane (Lai et al., 2016). The lower amounts of CdtC and CdtB in the *tlyA* mutant (**Figure 1B**) correlate with reduced ability of *C. jejuni* to attach and invade host cells (**Figure 3**), and is consistent with previous reports of CDT-production by *Campylobacter* strains stimulating adherence to and invasion of epithelial cells (Biswas et al., 2006; Ghorbanalizadgan et al., 2018).

After inactivation of the *cdtC* gene, we observed reduced adherence (**Figure 3A**) and host cell invasion by *C. jejuni* (**Figure 3B**), but to a lesser extent than for the *tlyA* mutant. While confirming the involvement of CDT in these processes, this at the same time emphasizes that other factors are involved. CdtC and the other CDT components have been detected in the *C. jejuni* cytoplasmic and the periplasmic space, although they are mainly associated with its OMVs (Lindmark et al., 2009; Elmi et al., 2012), suggesting that CDT localization within the bacterium is of importance for toxin delivery. OMVs play key roles in bacterial physiology and pathogenesis, ranging from secretion and delivery of biomolecules (including toxins, DNA, and quorum sensing molecules), stress response, biofilm formation, adherence to host cells, and immunomodulation (Roier et al., 2016). Consistent with this, the adhesion and invasion properties of *C. jejuni* can be enhanced by adding extra purified OMVs to the epithelial cell incubation mix (Elmi et al., 2012; Taheri et al., 2018). The addition of purified OMVs to our *C. jejuni*/Caco-2 cell incubation mixtures showed that while CdtC is important for host attachment (**Figure 4A**), it appears to play a greater role in host invasion (**Figure 4B**).

Prior to epithelial cell invasion, *C. jejuni* elicits an immune response and, in mice, the pathogen is quickly eliminated if its CDT activity is lacking (Fox et al., 2004). Here, we tested our *C. jejuni* mutants and their OMVs in an *in vitro* system using T84 epithelial cells assaying for induction of IL-8 release as a proxy for an innate immune response (Hickey et al., 1999; Hickey et al., 2000). The absence of CdtC in purified OMVs reduced their ability to induce IL-8 production (**Figure 4C**), from which we would infer that without CdtC the entry of the main actor, the CdtB toxin, is not facilitated. OMVs from the *tlyA* mutant (in which CdtC is present, albeit in reduced amounts) were as effective as wild-type OMVs at stimulating IL-8 release, suggesting that the smaller amount of CdtC facilitates entry of sufficient CDT to provoke the immune response. Interestingly, the lowest IL-8 immune response was seen in the case of the OMVs from cells in which the *mla* operon had been inactivated.

The *mla* operon encodes three proteins: a permease, MlaE; an ATP-binding protein, MlaF; and periplasmic substrate-binding, MlaD (Roier et al., 2016). The Mla (maintenance of lipid asymmetry) proteins were initially proposed to traffic phospholipids from the inner membrane (IM) to the outer membrane (OM) and are involved in formation of OMVs for the transport of lipids, cholesterol and other steroids (Ekiert et al., 2017). However, more recent work on Mla in *Acinetobacter baumannii* supports an alternative model for the directionality of the Mla system being exclusively retrograde (i.e. from the OM to IM) (Powers and Trent, 2019; Powers et al., 2020). It has been proposed that downregulation or mutation of the *mla* genes can lead to accumulation of phospholipids in the outer leaflet of the OM, which could promote OMV formation (Roier et al., 2016). This fits with the increase in OMV production observed here in the *ΔmlaEFD* strain (**Table S5**), which has also been reported upon *mlaA* mutation (Davies et al., 2019). Despite these effects, Mla proteins are apparently absent within *C. jejuni* OMVs (Elmi et al., 2012).

Inactivation of *tlyA* leads to a decrease in the abundance of all three MlaE, MlaF and MlaD proteins (**Figure 2**), indicating that their expression was being coordinately downregulated. Downstream of the *mla* operon, expression of *cj1640* was also reduced, suggesting its expression is linked to that of *mlaEFD*. Although the role of protein CJ1640 has not been determined, it shows similarity to proteins linked with cholesterol transport, and bears a vague resemblance to MlaB, a protein that has been reported to be absent in *Helicobacter pylori* (Roier et al., 2016), which has recently been reassigned with *C. jejuni* to the class of *Campylobacteria* (Waite et al., 2017; Parks et al., 2018). Reduced expression in this chromosome region is limited to the genes extending from *mlaE* to *cj1640*: expression of *tkt* (situated immediately upstream) and *cj1641* (immediately downstream) remained unaffected (**Figure 2**). Upon inactivation of the entire *mla* operon we observed a minor decrease in *C. jejuni* adherence and invasion of host cells (**Figure 3**) although, unexpectedly, OMVs from the Δ*mla* strain promoted these abilities in wild-type *C. jejuni* cells (**Figure 4**). Sensitivity to polymyxins was increased in the Δ*mla* strain (**Table S4**), suggesting a weakening of the OM structure that was not apparent in the Δ*tlyA* strains where the Mla proteins were still present, albeit in reduced amounts (**Table 3**). Tolerance to vancomycin, an inhibitor of cross-linking of the peptidoglycan matrix, remained unaffected by loss of *mlaEFD* (**Table S4**). This would point to the role of the Mla proteins being confined to the OM, and fits with the reduced ability of Δ*mla* OMVs to stimulate IL-8 production (**Figure 4C**). Counterparts of the MlaEFD proteins are present in *Haemophilus influenzae* (Davies et al., 2019), and have been shown to possess similar properties, with their loss causing attenuation of virulence (Fernández-Calvet et al., 2018).

The ability of *C. jejuni* cells to form biofilms and to adhere to abiotic surfaces in food-processing systems, and later to biotic surfaces, is essential for their transmission and ability to cause disease (Walan and Kihlström, 1988; Nguyen et al., 2011; Burnham and Hendrixson, 2018). Biofilm formation was extensively disrupted in the Δ*tlyA* strain (Sałamaszyńska-Guz et al., 2018), and the lack of such an effect with the Δ*mlaEFD* or Δ*cdtC* strains indicates that other cellular components are playing the prominent role in this process. One such candidate would be LptA, found in the periplasm of many Gram-negative bacteria where it transports lipopolysaccharides (LPS) and lower molecular weight lipooligosaccharides (LOS) for OM biogenesis (Ruiz et al., 2009). Depletion of LptA after *tlyA* inactivation (**Table S2**) would thus reduce the strain’s ability to maintain the asymmetry of the outer leaflet of the OM, altering cell surface hydrophobicity and attachment to host cells (Walan and Kihlström, 1988; Nguyen et al., 2011). Interrupting the delivery of LOS to the OM reduces *Campylobacter* invasion of intestinal epithelial cells *in vitro* (Kanipes et al., 2008) and *in vivo* in mice (Naito et al., 2010) and chickens (Iwata et al., 2013). The observations fit with studies on *Acinetobacter baumannii* where a deficiency of LOS was shown to reduce both growth and virulence (May and Grabowicz, 2018).

In conclusion, comparison of the *tlyA* knockout with the wild-type strain showed about fifty significant changes in protein abundance, over half of which were upregulated (**Table S2**). More stringent criteria for judging the effects of loss of TlyA methylation were applied by comparing the isogenic strains Δ*tlyA*::*tlyA*K188A and Δ*tlyA*::*tlyA*, and indicated that key changes causing loss of virulence are linked to fewer proteins, all of which are downregulated. The genes encoding four of these proteins (CdtC, MlaE, MlaF and MlaD), which were consistently downregulated in all TlyA^+^ and TlyA^-^ strain comparisons (**Table 3**), were themselves inactivated to test the principle of using an rRNA methyltransferase deficient strain as a means of identifying factors specifically required for virulence. We infer from the *cdtC* strain that CdtC plays a role in the host cell invasion process (**Figure 3B**). Inactivation of the *mlaEFD* operon confirmed the involvement of the Mla proteins in maintaining the integrity of the OM and reducing cell fragility under agitated culturing conditions, and these proteins also play a role in prompting the IL-8 innate immune response (**Figure 4C**).

We emphasize that these proteins (**Table 3**) by no means constitute a comprehensive list of factors involved in *C. jejuni* pathogenesis. Other cell components, including for example CdtB and LptA (**Tables 2** and **S3**) that were downregulated to a slightly lesser degree in TlyA^-^ strains, also would play important roles. However, we believe that we can conclude from these initial proteomics analyses that attenuating virulence by modulating ribosome function is a useful tool for defining key virulence factors in *C. jejuni*, and is an approach which might be applied to other bacterial pathogens.

## Data Availability Statement

The datasets presented in this study can be found in online repositories. The names of the repository/repositories and accession number(s) can be found below: http://www.proteomexchange.org/, PXD030125.

## Author Contributions

AS-G designed the experiments. AS-G, PKR, and MM performed the experiments. AS-G data analysis. SD and AS-G data interpretation. SD and AS-G: writing, review and editing. All authors contributed to manuscript revision, read, and approved the submitted version.

## Funding

Support from the National Science Centre [2018/30/M/NZ6/00429] to AS-G, and from the Danish Research Agency (FNU-rammebevilling 10-084554) to SD is gratefully acknowledged.

## Conflict of Interest

The authors declare that the research was conducted in the absence of any commercial or financial relationships that could be construed as a potential conflict of interest.

## Publisher’s Note

All claims expressed in this article are solely those of the authors and do not necessarily represent those of their affiliated organizations, or those of the publisher, the editors and the reviewers. Any product that may be evaluated in this article, or claim that may be made by its manufacturer, is not guaranteed or endorsed by the publisher.
