# Supplementary material for: Campylobacter jejuni Virulence Factors Identified by Modulating Their Synthesis on Ribosomes With Altered rRNA Methylation
Source: Front Cell Infect Microbiol. 2022 Jan 13;11:803730. doi: 10.3389/fcimb.2021.803730 (PMC8794745; doi:10.3389/fcimb.2021.803730)
Supplement: Supplementary file 1 [file DataSheet_1.docx]

**Supplementary Information**

***Campylobacter jejuni* virulence factors identified by modulating their synthesis on ribosomes with altered rRNA methylation**

**Agnieszka Sałamaszyńska-Guz,^1*^ Pernille Kronholm Rasmussen,^2^ Małgorzata Murawska,^1^ and Stephen Douthwaite^2*^**

^1^ Division of Microbiology, Department of Pre-Clinical Sciences, Institute of Veterinary Medicine, Warsaw University of Live Sciences – SGGW, Ciszewskiego 8, 02-786 Warsaw, Poland

^2^ Department of Biochemistry and Molecular Biology, University of Southern Denmark, Campusvej 55, DK-5230 Odense M, Denmark.

**Running title:** Identification of *Campylobacter jejuni* virulence factors

**Key words:** *Campylobacter jejuni*, rRNA methylation, *tlyA*, OMV, *mlaEFD*, CDT,

^*^Correspondence to:

Agnieszka Sałamaszyńska-Guz: [agnieszka_salamaszynska_guz@sggw.edu.pl](mailto:agnieszka_salamaszynska_guz@sggw.edu.pl)

Tel +48 22 593 60 30, Fax: +48 22 593 60 662

Stephen Douthwaite: [srd@bmb.sdu.dk](mailto:srd@bmb.sdu.dk); Tel +45 6550 2395

| **Primer** | **Primer sequence (5´ – 3´)** |
| --- | --- |
| cdtCR | TTATTCTAAAGGGGTAGCAGC |
| cdtCF | ATGAAAAAAATTATTACTTTGTTTTTTATGTTT |
| 1637F | ATGAATGCAAATTTCAAATTTCAAAATAAC |
| 1639R | TTATTTTTCTCCTGGTCCTAGTTTAGGTTTG |
| cdtC_mutR | CTTCTAAGATGGCTAAACAAAGATCGC |
| cdtC_mutF | GGAGCAAAATCTTGTCAAGATGATC |
| 1637_mutR | GCCTGATAGGCTAAAACCACTCCTAC |
| 1639_mutF | TTTGGTTAAAACGGCCGATGATAAATC |

**Table S1. Oligonucleotide primers used for cloning and mutagenesis.**

| ***C. jejuni* strains** | **TlyA protein** | **23S rRNA**  **nucleotide C1920 2´-*O*-methylation** | **Pathogenecity traits** | | | |
| --- | --- | --- | --- | --- | --- | --- |
|  |  |  | **biofilm**  **formation** | **cell adhesion** | **cell invasion** | **induction of IL-8**  **secretion** |
| **81-176** | active | present | +++ | +++ | +++ | +++ |
| **Δ*tlyA*** | none | none | ++ | + | + | ++ |
| **Δ*tlyA*::*tlyA*** | active | present | +++ | +++ | +++ | +++ |
| **Δ*tlyA*::*tlyA***  **K188A** | inactive | none | ++ | + | + | ++ |

**Table S2. Pathogenic characteristics of the *C. jejuni* *tlyA* (*cj0588*) mutants.**

The pathogenic traits are graded with a plus system where + signifies 0 – 40 % of the wild-type ability; ++ 41-80 %; and +++ > 81 %.

**Table S3 text**

Table S3 is presented as an Excel file and lists all the significant changes in protein abundance in the comparisons of TlyA^+^ (wild-type, WT and Δ*tlyA*/Δ*tlyA*::*tlyA*) with TlyA^-^ strains (Δ*tlyA* and Δ*tlyA*::*tlyA*K188A). In the Δ*tlyA* strain, the *tlyA* gene has been inactivated by insertion of a chloramphenicol resistance cassette; Δ*tlyA*::*tlyA* is complemented with an active copy of *tlyA* inserted into the chromosome at the 121-bp intergenic region between cj0652 and cj0653c loci; Δ*tlyA*::*tlyA*K188A is isogenic with Δ*tlyA*::*tlyA* and differs only at the *tlyA*-188 locus where a single-site mutation at the catalytic site renders the TlyA methyltransferase inactive (Sałamaszyńska-Guz *et al.,* 2018). Column A lists the protein accession numbers in UniProt; column B describes the protein that are changed; column C gives the gene ID numbers in the *Campylobacter jejuni* 81-176 strain and in this notation, *tlyA* is CJJ81176_0616. The expression of genes upstream (e.g. CJJ81176_0615, encoding a putative membrane protein) and downstream (e.g. CJJ81176_0617, the riboflavin biosynthesis protein, RibF, and CJJ81176_0618, a putative tRNA methyltransferase) were not significantly changed in the Δ*tlyA* strain. We conclude from this that there is no indication of polar effects arising from insertion of the *cat* resistance cassette. Columns D to G compare the TlyA^+^ with TlyA^-^ strains where downregulated proteins with a fold change ≥ 1.5 are marked in green, and upregulated proteins with a fold change ≥ 1.5 are marked in red; columns H to K give the same data as log_2_ values; columns L to O give the P values for two technical replicates for each of three independent biological replicates; the following columns show the raw data for these individual replicates.

| ***C. jejuni* strains** | **Polymyxin E**  **(mm)** | **Polymyxin B**  **(mm)** | **Vancomycin**  **(mm)** |
| --- | --- | --- | --- |
| Wild-type | R | 15 | R |
| Δ*tlyA* | R | 15 | R |
| Δ*mlaEFD* | 13 | 19 | R |

**Table S4. Susceptibilities of the *C. jejuni* 81-176** **wild-type strain and its derivatives to antimicrobial agents determined by disc diffusion.**

The *C. jejuni* strains were grown under microaerobic conditions at 37 °C on MH agar containing 5 % (v/v) sheep blood. The inhibition growth zones are given in mm. Drug concentrations on discs: polymyxin E (10 μg), polymyxin B (300 μg) and vancomycin (30 μg). R, resistant (no inhibition of growth around the disc).

| ***C. jejuni* strain** | **OMV produced (μg/ml culture)** |
| --- | --- |
| Wild-type | 107 ± 7.5 |
| *ΔtlyA* | 125 ± 22 |
| *ΔcdtC* | 101 ± 2 |
| *ΔmlaEFD* | 140 ± 25* |

**Table S5. Production of OMVs by *C. jejuni* strain 81-176 (wild-type) and the *tlyA, cdtC* and *mlaEFD* knockout derivatives of this strain.**

The values are the means and standard deviations from measurements of three independent cultures for each strain. *The amount of OMVs in the *ΔmlaEFD* strain is significantly higher than in the wild-type (P < 0.05); OMV production in the *ΔtlyA*and *ΔcdtC*strains was not significantly different from the wild-type.
